# Involvement of serotonergic receptors in depressive processes and their modulation by β-arrestins: A review

**DOI:** 10.1097/MD.0000000000038943

**Published:** 2024-07-12

**Authors:** Aldo R. Tejeda-Martínez, Ana R. Ramos-Molina, Patricia A. Brand-Rubalcava, Mario E. Flores-Soto

**Affiliations:** a Laboratorio de Neurobiología Celular y Molecular, División de Neurociencias, Centro de Investigación Biomédica de Occidente (CIBO), Instituto Mexicano del Seguro Social, Guadalajara, México; b Departamento de Ingeniería Química, Centro Universitario de Ciencias Exactas e Ingenierías, Universidad de Guadalajara, Guadalajara, México.

**Keywords:** 5-HT receptors, depression, serotonin, β-arrestin

## Abstract

Over time, several studies have been conducted to demonstrate the functions of the neurotransmitter 5-hydroxytryptamine (5-HT), better known as serotonin. This neurotransmitter is associated with the modulation of various social and physiological behaviors, and its dysregulation has consequences at the behavioral level, leading to various neurophysiological disorders. Disorders such as anxiety, depression, schizophrenia, epilepsy, sexual disorders, and eating disorders, have been closely linked to variations in 5-HT concentrations and modifications in brain structures, including the raphe nuclei (RN), prefrontal cortex, basal ganglia, hippocampus, and hypothalamus, among others. The involvement of β-arrestin proteins has been implicated in the modulation of the serotonergic receptor response, as well as the activation of different signaling pathways related to the serotonergic system, this is particularly relevant in depressive disorders. This review will cover the implications of alterations in 5-HT receptor expression in depressive disorders in one hand and how β-arrestin proteins modulate the response mediated by these receptors in the other hand.

## 1. Introduction

Serotonin (5-HT) is a neurotransmitter associated with the regulation of various physiological functions, such as body temperature, appetite, sexual desire, the sleep-wake cycle, memory, learning, emotions, and mood regulation.^[1]^ The effect of 5-HT is modulated by the action of 14 serotonergic receptors, grouped into 7 subfamilies: 5-HT_1_, 5-HT_2_, 5-HT_3_, 5-HT_4_, 5-HT_5_, 5-HT_6_, and 5-HT_7._ Except for the ionotropic receptor 5-HT_3_, these receptors are of the metabotropic type coupled to G proteins.^[2]^ The system also includes the monoamine oxidase A degradative enzyme (MAO-A) and the selective 5-HT transporter (SERT).^[1]^ Due to their relationship in mood modulation, alterations in these components of serotonergic neurotransmission have been implicated in disorders such as depression.

Depression is a mood disorder with varied symptoms. It is mainly characterized by irritability, sadness, and anhedonia, accompanied by cognitive and physiological alterations, including neurodegeneration. This disorder implies a significant deterioration in the quality of life of those who suffer from it.^[3]^ The heterogeneity in depression symptomatology suggests that various brain structures, including the hippocampus, basal ganglia, septum, raphe nuclei (RN), amygdala, nucleus accumbens, and prefrontal cortex (PFC), exhibit morphophysiological alterations and changes in the density of serotonergic receptors.^[4,5]^

On the other hand, cytoplasmic β-arrestin proteins participate in the desensitization and internalization of serotonergic receptors into the cytoplasm, thereby modulating receptor functionality and serotonin-mediated signaling.^[6]^ However, the involvement of β-arrestins is not limited to endocytosis, they can initiate a signaling cascade in signal transduction. β-arrestin could translocate to the nucleus, promoting gene expression by binding to transcription cofactors, such as cyclic adenosine monophosphate (cAMP) response element-binding (CREB), or participating in the ubiquitination of transcription factors.^[7]^ The involvement of β-arrestins in cell proliferation and hippocampal neurogenesis has also been implicated, suggesting their role as regulators of the signal through which fluoxetine (FLX) exerts its antidepressant effects.^[8,9]^

Therefore, the aim of this review is to explore the role of β-arrrestins in mediating depression by modulating 5-HT receptor signaling.

### 1.1. Serotonergic system

The serotonergic system comprises the monoaminergic neurotransmitter 5-HT, and its functionality is mediated by postsynaptic receptors, autoreceptors, the SERT, and the 5-HT catalytic enzyme. 5-HT synthesis primarily occurs in the neurons of the RN at the brain level. This synthesis occurs from the essential amino acid l-tryptophan, catalized by the enzyme tryptophan hydroxylase, mainly isoform 2 (TPH2).^[10]^ Tryptophan is then hydroxylated to 5-hydroxytryptophan (5-HTP), and subsequent decarboxylation of 5-HTP generates 5-HT. The latter is then recaptured by the vesicular monoamine transporter in synaptic vesicles to prevent degradation. Subsequently, mediated by the action potential, 5-HT is released into the synaptic cleft^[10,11]^ and distributed throughout the central and peripheral nervous system (central nervous system). The 5-HT available in the synaptic space is recaptured by SERT for reuse.^[12]^ Finally, 5-HT undergoes catabolism by the enzymatic action of MAO-A, resulting in the major reduced metabolite of 5-HT, 5-hydroxyindole-acetic acid,^[13]^ for subsequent excretion in urine conjugated with glucuronic acid.^[14]^

As mentioned above, the RN are a set of 6 neuronal nuclei grouped in pairs and located in the midline of the brainstem, along the midbrain. Predominantly composed of serotonergic neurons, these nuclei project to various cortical and subcortical areas of the brain. The RN play a crucial role in functions related to the sleep-wake cycle, response to stimuli, and the synthesis and release of 5-HT.^[15]^ Consequently, changes in RNs’ neuronal functions decreased serotonergic activity, and increased expression of autoreceptors have been associated with various neurological and behavioral disorders, including depression.^[16,17]^

## 2. Variations in the components of the serotonergic system

### 2.1. Autoreceptors and heteroreceptors

At the brain level, most neurons, whether serotonergic or not, are modulated by the action of this neurotransmitter, as serotonergic receptors are present in the majority of brain cells. On the other hand, because RN project terminals to various subcortical regions, such as the hippocampus, amygdala, hypothalamus, thalamus, and different areas of the prefrontal cortex, 5-HT modulates various behavioral and neural patterns as needed.^[18]^ Following the synthesis, storage, and release of the neurotransmitter 5-HT, it interacts in the synaptic cleft with both pre- and postsynaptic receptors. In this regard, at least fourteen 5-HT receptor subtypes have been identified in the mammalian brain, which have been grouped into seven families^[2]^ see Table 1.

**Table 1:** 5-HT receptor classification.

5-HT_1_	5-HT_2_	5-HT_3_	5-HT_4_	5-HT_5_	5-HT_6_	5-HT_7_
5-HT_1A_ 5-HT_1B_ 5-HT_1D_ 5-HT_1E_ 5-HT_1F_	5-HT_2A_ 5-HT_2B_ 5-HT_2C_	5-HT_3_	5-HT_4_	5-HT_5A_ 5-HT_5B_	5-HT_6_	5-HT_7_

**Figure 1.**
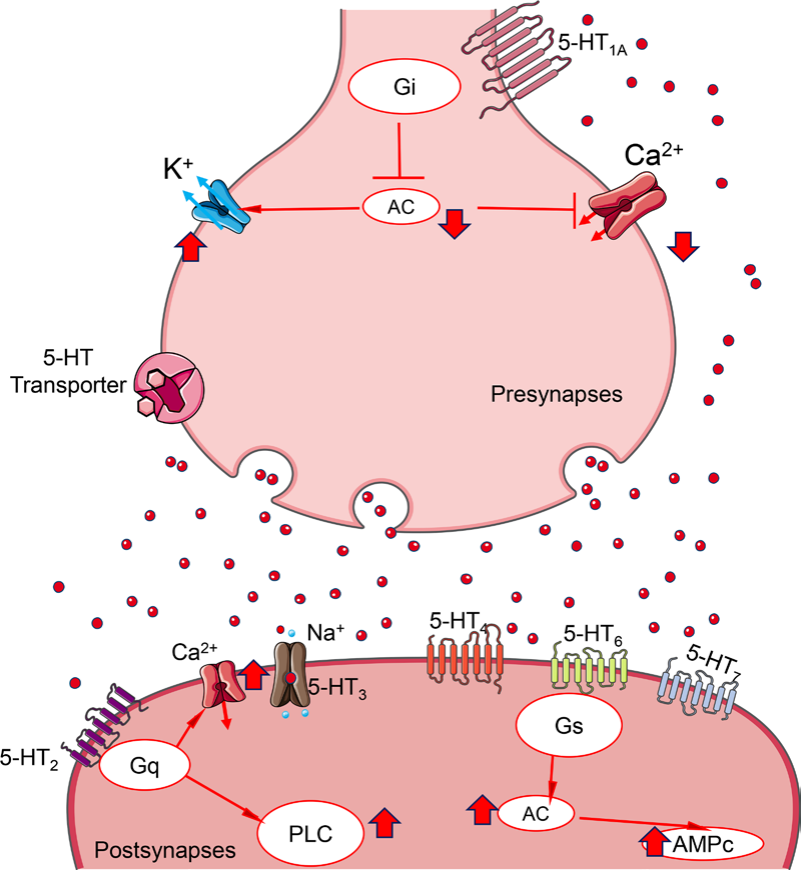
5-HT synthesis and serotonergic synapse. 5-Hydroxytryptamine (5-HT) is synthesized from tryptophan by the enzyme tryptophan hydroxylase and is stored in vesicles by VMAT for subsequent release into the synaptic space. 5-HT receptors are found in both the presynaptic neuron (autoreceptors) and postsynaptic neuron (heteroreceptors). By binding to G proteins, these receptors exert their action. AC = adenyl cyclase, cAMP = cyclic adenosine monophosphate, PLC = phospholipase C.

G protein-coupled receptors consist of seven transmembrane domains, and their action and function are modulated by the G protein to which they are coupled (Fig. 1). Depending on their location, serotonergic receptors on neurons can function as autoreceptors (presynaptic) or heteroreceptors (postsynaptic). On the one hand, the 5HT_1_ receptors inhibit neuronal firing by activating K^+^ channels and inhibiting Ca^2+^ channels.^[19]^ They also promote the inhibition of pyramidal neurons in the hippocampus.^[20]^ Meanwhile, 5HT_2_ receptors activate Ca^2+^ channels.^[21]^ Finally, the receptors 5-HT_4_, 5-HT_6_ Y 5-HT_7_ increase cAMP, thereby enhancing neuronal excitability^[20]^ (Fig. 1).

On the other hand, depressive disorders have been closely linked to changes in the expression, affinity, or coupling of 5-HT_1A_ and 5-HT_2A_ receptors, both heteroreceptors and autoreceptors. These receptors are widely distributed throughout the brain. In the case of 5-HT_1A_ autoreceptors, they are located in the RN and innervate serotonergic connections to various cortical and subcortical areas. While heteroreceptors are mostly found in cortical and subcortical regions, the cerebral cortex is one of the brain areas with the greatest innervation of serotonergic afferents, which is why its dysregulation is extensively implicated in depressive disorders.^[4,5]^

Studies indicate that activation of serotonin receptor subtypes 5-HT_1A_ and 5-HT_2A_ are responsible for mediating the excitability of certain neurons, particularly, layer V pyramidal neurons. The injection of endogenous 5-HT into cortical areas influences the activation of serotonergic receptors in both pyramidal and nonpyramidal neurons, contributing to excitatory and inhibitory neurotransmission through the activation of subcortical circuits.^[22]^ Preclinical studies suggest that activation of postsynaptic 5-HT_1A_ receptors in layer V pyramidal neurons promotes neuronal membrane hyperpolarization, resulting in inhibition of neurotransmission. Conversely, when 5-HT_2A_ receptors are activated, they mediate neuronal excitability by depolarizing this neuronal pool and consequently promoting the activation of gamma-aminobutyric acid (GABA)ergic interneurons.^[23]^

### 2.2. 5-HT1 receptors

Serotonergic autoreceptors, including 5-HT_1A_, 5-HT_1B_, and 5-HT_1D_ subtypes located in the membranes or presynaptic somatodendritic zones of serotonergic neurons in RN,^[24–26]^ primarily regulates 5-HT synthesis and release into the synaptic space.^[27]^ Their main mechanism of action involves negative feedback, through constant stimulation by 5-HT recaptured by SERT. These autoreceptors inhibit the firing of 5-HT into the synaptic space, making them a pharmacological target for antidepressant treatments such as selective serotonin reuptake inhibitors (SSRIs).^[27]^

When SERT is inhibited, 5-HT feedback to the presynaptic neuron decreases, leading to internalization of 5-HT_1A_ autoreceptors and subsequently increasing serotonergic discharge.^[28]^ Several studies have demonstrated that activation of 5-HT_1A_ autoreceptors through the administration of agonists, such as 8-hydroxy-2-(di-N-propylamino) tetralin (8-OH-DPAT), decreases the density of autoreceptors in the RN membrane leading to increased 5-HT discharge into the synaptic space.^[29]^

As Gαi/o protein-coupled receptors, the main activation pathway of the 5-HT_1_ subfamily involves the inhibition of adenyl cyclase (AC) and cAMP synthesis. Upon activation of 5-HT_1_ receptor-coupled proteins, the initial response is the exchange of guanosine diphosphate for guanosine triphosphate which stimulates the dissociation of the trimeric complex to alpha-guanosine triphosphate and beta-gamma subunits, leading to the inactivation of AC. This inactivation of said enzyme results in decreased concentrations of cAMP on the cytoplasm, leading to a diminishment of protein kinase A (PKA) activity, leading to blockade of Ca^2+^ – but not K^+^ – ion-dependent channels. This signaling cascade induces depolarization of the neuronal membrane. In the case of RN autoreceptors, it results in decreased 5-HT release, while the activation of heteroreceptors decreases neuronal excitability.^[21,29–31]^

Serotonergic heteroreceptors are mostly expressed in non-serotonergic interneurons, and their activation mediates the release of different neurotransmitters. Within the limbic system, these heteroreceptors are located in the somatodendritic zone of glutamatergic interneurons, mediating, with their activation, the release of the neurotransmitter glutamate.^[32]^ In the axons of cholinergic and GABAergic interneurons, these heteroreceptors modulate the action of acetylcholine and GABA, respectively.^[33–35]^

Whitin the midbrain, they modulate dopamine release, and in the prefrontal cortex, they modulate glutamate release.^[32,36]^ This indicates that the action of autoreceptors differs not only from that of heteroreceptors, but also across different brain areas. As reported by Riad et al,^[29]^ using electron microscopy, intravenous administration of the agonist 8-OH-DPAT decreases the density of 5-HT_1A_ autoreceptors in the membrane of RN dendrites at 15 minutes and 1 hour. This reduction led to the internalization of these receptors, leading to increased 5-HT firing. In contrast, in the case of hippocampal 5-HT_1A_ heteroreceptors, there was no change in density. Meanwhile, 24 hours after 8-OH-DPAT administration, 5-HT_1A_ autoreceptors returned to basal levels. This effect was also observed to be prevented by WAY100635, a 5-HT_1A_ autoreceptor antagonist.^[29]^ Similarly, it has been suggested that the regulation of autoreceptors in RN is central to the mechanism of action of antidepressants as they regulate the release of 5-HT, along with the activation of 5-HT_2A_ receptors and other neurotransmitters. In this sense, it has been corroborated that the overexpression of 5-HT_1A_ autoreceptors in mice presents an increase in neuronal inhibition and a depressive phenotype, as well as a resistance to SSRIs.^[37]^ This highlights the differentiation in the functionality of each of these, differing in their localization.

Lastly, 5-HT_1E_ and 5-HT_1F_ are among the less studied receptors, in part due to their absence in mice and the challenge of assigning them a specific role in particular physiological process or diseases. Nevertheless, their presumptive presence in frontal cortex and hippocampus suggest that they might be considered as relevant drug targets in the future.^[38]^

### 2.3. 5-HT_2_ receptors

5-HT_2A_ receptor subtypes at the cortex level are widely distributed in apical dendrites of GABAergic interneurons and serotonergic neurons in somatodendritic areas of sensory and motor neurons. Whereas, in other brain areas, they are located in the RN, hippocampus, amygdala, thalamus, and hypothalamus.^[39]^ The 5-HT_2A_ receptor, being widely distributed in cortical and subcortical areas, has been detected in GABAergic interneurons. Its main effect upon activation is an inhibitory postsynaptic potential.^[2]^ The administration of various doses of 5-HT_2A_ receptor agonists, such as 2,5-Dimethoxy-4-iodoamphetamine (DOI), increases GABA neurotransmitter concentrations in pyramidal neurons of the PFC, and the administration of antagonists attenuate 5-HT firing by activation of GABAergic inhibition.^[22,40,41]^ On the other hand, 5-HT_2A_ receptors have been closely related to the phenotype of depressive disorders. Over time, the administration of antidepressant treatments decreases the expression of 5-HT_2A_ receptors. Additionally, the blockade of these receptors by their antagonist M100907 increases the efficacy of antidepressant drugs such as SSRIs and shows antidepressant-like effects,^[42,43]^ suggesting that the activation of 5-HT_2A_ receptors produces favorable effects for the treatment of depression.

In addition to serotonergic neurons, GABAergic interneurons are located in large concentrations in RN, although they are mainly distinguished by their distribution in different locations and their pharmacological response. Similarly, GABAA and GABAB GABAergic receptors are expressed in serotonergic neurons.^[44,45]^ In RNs, GABAergic interneurons have the main function of regulating the excitatory response of serotonergic neurons, thus controlling the release of 5-HT to different brain areas.^[46]^ The 5-HT_2A_ subtype receptors respond through the activation of second messenger cascades. As Gαq protein-coupled receptors, they activate phospholipase C beta (PLC) resulting in the hydrolysis of phosphatidylinositol bisphosphate which generates inositol triphosphate (IP3) and diacylglycerol. This could lead to the opening of IP3 gated channels on the endoplasmic reticulum to increase intracellular calcium. A second pathway of action is through AC synthesis, increasing cAMP and thus overexpression of brain-derived neurotrophic factor brain derived neurotrophic factor.^[39]^

## 3. The role of 5-HT_1A_ and 5-HT_2A_ receptors in depressive disorders

There have been several studies reported at both preclinical and clinical levels on the involvement of serotonergic receptors in depressive disorders, facilitated by the use of agonist drugs, antagonists, imaging studies, genetic modification, and postmortem studies. These investigations aim to obtain a better understanding of the participation of different components of the serotonergic system in depression, with the goal of proposing more effective treatments to alleviate the symptoms of this disorder.

The direct involvement of 5-HT receptors has been extensively investigated to clarify their role in the genesis of depression and understand the mechanism through which each of these receptors is related. The possibility of modifying or improving specific drugs to treat this disorder is also crucial. In this regard, reports indicate that chronic treatment with FLX (a drug used to counteract depressive symptoms, belonging to the SSRI family) desensitizes 5-HT_1A_ heteroreceptors in the hypothalamus, an effect dependent on serotonergic innervation.^[47]^ Preclinical and clinical studies – imaging and postmortem – have demonstrated the correlation of the expression, activation, and signaling of 5-HT_1A_ and 5-HT_2A_ receptors with depressive disorder.^[25,35,48–50]^

In fact, the relationship between these two types of receptors and depressive disorders is such that the differences of the expression of both 5-HT_1A_ and 5-HT_2A_ have been studied in relation to antidepressant effects of different drugs listed in the Table 2 below:

**Table 2:** Effects of antidepressants on the 5-HT_1A_ and 5-HT_2A_ expression.

Experimental subject	Treatment	Experimental model	Identified effect	Ref.
Adult male Wistar rats	Fluoxetine (FLX) was administered with drinking water at a dose of approximately 7.5 (7.26–7.70) mg/kg/day for 8 weeks	Forced swim test (FST)	It increased 5-HT_1A_ receptor expression in FST in the hippocampus, and prefrontal cortex in control rats, while FLX administration significantly decreased receptor expression in the prefrontal cortex	Shishkina et al (2012)
Sprague-Dawley male rats	Escitalopram (ESC) and Aripiprazole (ARI) 10 and 2 mg/kg/day, respectively, via osmotic or cumulative injections.WAY-100635 (WAY; 0.01–1 mg/kg) and 8-OH-DPAT (DPAT; 0.3–1 mg/kg)	Forced swim test	ESC + ARI administration increased 5-HT_1A_ receptor mRNA expression in the hippocampus, an effect reversed by 8-OH-DPAT agonist administration. On the other hand, in the raphe nuclei, a decrease in receptor mRNA expression was observed in all treatments with respect to the control. In the case of FST, immobility time increased with ESC + ARI + WAY treatment and decreased with ESC + ARI + DPAT	Lapointe et al (2019)
Sensitive line (FSL) male rats of Sprague-Dawley (SD)	5 µM 8-OHDPAT (i.c.v) 10 µM SUN11602 (i.c.v) FGF2 10 ng/mL (i.c.v)	Forced swim test	Flinder’s sensitive line rats (FSL) treated with 8-OHDPAT decreased immobility time compared to the control. Administration of the agonist SUN11602 and FGF2 reduced the K + currents induced in the CA1 area of the hippocampus. The combination of 8-OH-DPAT and SUN11602 agonists increased the density of the FGFR1-5-HT_1A_ heteroreceptor complex	Borroto-Escuela et al (2017)
Male congenital learned helpless and noncongenital learned helpless rats (generation 55)	Vehicle (100% DMSO), EMD 281014 (receptor antagonist 5HT_2A_) (0.03, 1, 3, 10 y 30 mg/kg, i.p 24, 5 and 1 hour before the test	Forced swim test	Decrease in immobility time with the 30 mg/kg dose	Patel et al (2004)
Fischer rats (intact control rats at random stages of the estrous cycle and ovariectomized rats for three months	17β-estradiol (E2), 10 µg b.i.d. s.c., twice daily	NE	Ovariectomized rats showed a 31% decrease in prefrontal cortex 5-HT_2A_ receptor density compared to intact rats. compared to intact rats. This effect was reversed by the E2 administration	Cyr et al (1998)
Balb/C mice	10 μg/kg 5HT_2A_ receptor antisense oligonucleotide every 12 hours for 4 days	Forced swim test	Decreased expression of the 5HT_2A_ receptor in the prefrontal cortex and striatum. Decrease in immobility time compared to the control	Sibille et al (1997)
postmortem study of healthy psychiatric patients and individuals who had a history of a major depressive episode.		NE	Patients with a history of major depressive episodes had lower 5-HT_2A_ receptor binding in the auditory cortex. Lower levels of SERT binding and higher levels of 5-HT_1A_ receptor binding were observed in the CPF	Steinberg et al (2019)
Adult male C57BL/6 J mice	acacetin or vehicle was administered to mice (v.g. 5, 15, and 45 mg/kg) for 21 consecutive days. After 3 weeks of acacetin or vehicle treatment, the mice were repetitively co-administered with WAY-100635, isamoltane, ritanserin, ondansetron or 8-OH-DPAT, 30 minutes following the acacetin dosing from day 21 to day 25	Forced swim test and Tail suspension test	Chronic treatment with 45 mg/kg acacetin decreases FST and TST immobility time similarly to the antidepressant drug FLX (16 mg/kg), without changes in locomotor activity, while 5-HT levels increase in the cortex and hippocampus. On the other hand, administration of the antagonist WAY-100635 (1 mg/kg) blocks the effect of acacetin on FST and TST. This suggests a serotonergic mechanism of action via 5-HT_1A_ receptor regulation of the anti-hopelessness-like activity of acacetin	Xiao et al, (2019)

5-HT_1A_ receptors are not limited to serotonergic neurons. As mentioned above, they act as autoreceptors modulating neurotransmitter discharge, and also as postsynaptic heteroreceptors in non-serotonergic neurons mediating inhibitory responses.^[46]^ Overall, it has been demonstrated that blocking or desensitization of 5-HT_1A_ autoreceptors elicits an antidepressant-like response by increasing 5-HT discharge. This is reflected in the modification of hopelessness-like behaviors in several tests, such as the forced swim test and tail suspension. Animals lacking the 5-HT_1A_ autoreceptor were observed to be less immobile than their controls, confirming the lack of signaling of this receptor as an antidepressant.

Concerning the 5-HT_2A_ receptor, although not extensively studied, is known that its response will depend on the brain area in which it is expressed. In this regard, a study was conducted in pregnant Sprague-Dawley rats in which the expression and behavioral response were examined by administering different doses (1, 2.5, or 5.0 mg/kg) of the selective agonist TCB-2 and the antagonist MK212 in the medial prefrontal cortex (mPFC), amygdala and RN. Results demonstrated that alterations in the expression of this receptor trigger depression or psychosis in the postpartum period.^[51]^ These receptors have also been linked to the stress-mediated response as a prelude to depression, as they regulate the expression of cortisol and adrenocorticotropic hormone. 5-HT_2A_ receptor agonists, such as quipazine, increase the concentration of these hormones.^[52]^ Studies in female and male mice lacking the 5-HT_2A_ receptor and exposed to a constant stressor modified the expression of stress response genes, corticosterone receptors, and BNDF in the prefrontal cortex and hippocampus with respect to the control, suggesting with these results a close relationship between stress regulation and the presence of 5-HT_2A_ receptors.^[53]^

Due to the close relationship of GABAergic receptors in the 5-HT-mediated synapse, several studies have been conducted to elucidate the role of serotonergic receptors on GABAergic receptors.^[23]^ In this regard, a preclinical model with mice lacking the GABAergic receptor explored the involvement of GABA_b1a_ and GABA_b1b_ receptors, mediated by the activation of the serotonergic 5-HT_1A_ receptor, by the evaluation of the selective 5-HT_1A_ receptor agonist (8-OH-DPAT) effect at a dose of 0. 5 mg/kg in the forced swim test. Authors observed a decrease in the immobility time in mice lacking the GABA_b1a_ subtype, suggesting that the blockade of these receptors can be linked to an anti-hopelessness effect. In addition, the administration of 8-OH-DPAT in mice lacking the GABA_b1a_ receptor resulted in a decrease in the hormone corticosterone.^[54]^

The use of natural treatments to counteract the effects of depression has focused on modulating the mechanism of action on these receptors. For example, the aqueous extract of *Tagetes lucida* Cav, was tested at different doses (50, 100, and 200 mg/kg) compared with 20 mg/kg of FLX (72, 48, 24, 18, and 1 hour before behavioral test) in male Wistar rats. Evaluation of its activity in the forced swimming test demonstrated an antidepressant-type effect by decreasing the total immobility time with the dose of 100 mg/kg, thereby enhancing the effect of FLX. Mechanistic exploration involved the use of various substances, such as WAY-100635 (0.5 mg/kg), a 5-HT_1A_ receptor antagonist, ketanserin (5 mg/kg), a 5-HT_2A_ receptor antagonist, propranolol (200 mg/kg), a β-noradrenergic receptor antagonist, and yohimbine (1 mg/kg), a α2-noradrenergic receptor antagonist. The study observed a suppression of the anti-hopelessness-like activity of the extract with the administration of WAY-100635 and ketanserin. This suggests that the anti-hopelessness-like effect of *Tagetes lucida* Cav extract is mediated by the interaction of serotonergic 5-HT_1A_ and 5-HT_2A_ receptors.^[55]^

Li and colaborators reported that sub-acute treatment with a curcumin derivative (J147 9 mg/kg v. g) had an anti-hopelessness-like effect in the forced swim test, reducing immobility time in a similar way to Imipramine (10 mg/kg). High affinity for the 5-HT_1A_ receptor was also observed using the radioligand assay. To corroborate the results, a 5-HT_1A_ receptor antagonist (NAD-299) was used, attenuating the effects of J147. Conversely, 8-OH-DPAT improved the effects of J147 in the behavioral test, along with the expression of BNDF, cAMP, PKA, and CREB, contributing to an increased anti-hopelessness-like effect.^[9]^

Noteworthy, subfamilies 4, 5, 6, and 7 have not been linked to depressive disorders. However, recent evidence suggest that the latter may exhibit differential activity depending on classical or β-arrestin mediated signaling pathway.^[56]^ The implications of these protein groups with 5-HT receptors and signaling will be further discussed.

## 4. β-arrestins and their role in the mechanism of action of serotonergic receptors

The arrestins are a family of cytoplasmic proteins with different isoforms (1, 2, 3, 4). They comprise two antiparallel β-sheet domains capable of recognizing phosphorylated residues of the active receptor. These sheets are connected through a hinge region and an α-helix at the N-terminus.^[57]^ Arrestin 2 and 3 (also named β-arrestin 1 and 2, respectively) are ubiquitously expressed, while arrestin 1 and 4 are expressed in retinal cones. β-arrestin 2, predominantly localized in the cytoplasm, exhibits a higher affinity for β-adrenergic, dopaminergic, and serotonergic receptors. The main function of β-arrestins is desensitization and internalization of G protein-coupled receptors, although they have also been shown to regulate β-arrestin-dependent intracellular signaling,^[58,59]^ thus leading to a differentiated G protein signaling or β-arrestin mediated signaling which in certain cases have been demonstrated independence of G protein signaling, although some of the second messengers within the cells are common for both like extracellular regulated kinases (ERK1/2). The generation of different responses according to different agonists although same receptor is activated is known as biased agonism by some authors^[56,60,61]^ meanwhile others have find the functional selectivity as an appropriate terminus for the same phenomena.^[62–64]^ Presumably most of the first examples for these types of differentiated responses to different agonist/antagonist are precisely from 5-HT receptor interactions, given its complex and diverse pharmacology.

## 5. β-arrestins role on internalization and relation with depressive disorders

Although review has been made about β-arrestin signaling and internalization on the receptors mainly described its role in depression remains little discussed although is a concept focusing the attention of diverse researchers lately.^[64,65]^

Treatment with fluvoxamine (SSRIs) decreases the expression of β-arrestin in the hippocampus, while imipramine and desipramine (tricyclics, SNRIs) increase it. This could be related to the type of drug and the redistribution of G proteins to achieve brain homeostasis.^[66]^ The involvement of β-arrestin2 has been suggested as part of the mechanism of action of antidepressant drugs, such as SSRIs. A significant increase in these proteins has been observed on day 21 of treatment with FLX in animal models of anxiety or depression induced by corticosterone administration.^[67]^ Meanwhile, mice lacking the gene encoding β-arrestin exhibited a reduced response to FLX treatment.^[8]^

Li et al,^[9]^ using a chronic stress model of depression, suggest that β-arrestin2 expression is a key component of the mechanism of action of FLX, as β-arrestin2 knockout mice do not respond to FLX treatment (10 mg/kg) on parameters such as immobility time, 5-HT concentrations, as well as hippocampal neurogenesis.

The expression and overexpression of various serotonergic receptors, mainly the 5-HT_1A/2A_ subtypes, have been implicated in the genesis of mood disorders. Actions of various drugs, over time, directly or indirectly modulate the expression of these receptors. For instance, chronic treatment with FLX, by inhibiting the action of SERT, leads to increased availability of 5-HT in the synaptic space and in the soma of the neuron. This mechanism might indirectly regulate the expression of 5-HT_1A_ autoreceptors. FLX has been reported to act as an agonist of serotonergic 5-HT_2B_ receptors in astrocyte cell lines lacking SERT. Continuous agonism tends to downregulate these receptors through internalization that could be conducted by β-arrestins or independently mediated.^[68]^

Similarly, a study by Avissar et al^[66]^ evaluated different antidepressants such as imipramine, desipramine, and fluvoxamine. Rats treated with fluvoxamine exhibited an increased expression of β-arrestin in the membrane of neurons from the cortex. For his part, imipramine and fluvoxamine treatment decrease the expression of β-arrestin in the membrane of hippocampal neurons. These results suggest an important association between β-arrestin expression and antidepressants, implicating that these proteins have an effect on the pharmacological response. Moreover, the importance of the internalization of serotonergic receptors, mediated by β-arrestins, underscores the significance of understanding this correlation to enhance the efficiency of antidepressant treatments.

Although, the complete mechanism by which serotonergic receptors are internalized could vary between each receptor, for most of them such processes remain unclear, as well as varying from experiments in vivo and in vitro, where several mechanisms of internalization have been identified for the same receptors in different culture cells. However, there is recent review regarding this issue in particular with 5-HT_1,2A_ pointing out the issue remains largely unknown.^[65]^ β-arrestins however have an important impact for both of these receptor subtypes activities due to their participation in Clathrin mediated endocytosis^[69]^ as well as other independent process of internalization not related to GRK2 and GRK5.^[70]^

**Figure 2.**
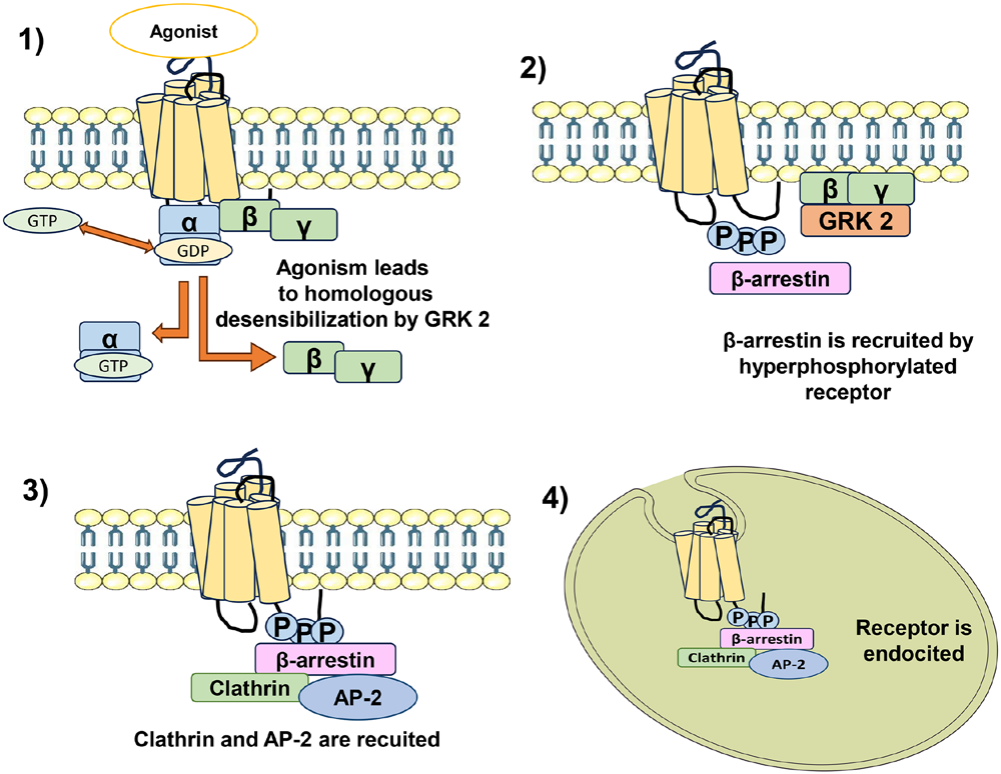
Mechanism of β-arrestin-mediated endocytosis of 5HT receptors. A) Starting with agonism which promotes the release of the trimer from G protein, thus allowing the phosphorylation of some residues near the C terminus of the receptor B) by G protein coupled receptor kinases (GRKs) GRK2 (GRK5 and GRK6 could play a role although been proved less efficient). Then this region acts as site for the union of β-arrestins2-3 which promotes Clathirn and other proteins recruitment C) and ultimately leading the endocytic process D). The agonism of a serotoninergic receptor could lead to internalization by this mechanism known as heterologous desensitization and could dampen the activity of the receptors up to 50%. Guanosine diphosphate, triphosphate GDP, GTP.

The binding of β-arrestin to G protein coupled receptors (GPCRs) could facilitate their endocytosis by the abovementioned mechanism (Fig. 2), although for some 5-HT subtypes of receptors it has been known that certain mechanisms of endocytosis occur in a β-arrestin independent manner.^[42,70]^ In the adult brain, β-arrestin2 is found in abundance in areas such as the hypothalamus, amygdala, and hippocampus.^[71]^ When an agonist like 5-HT binds to the corresponding GPCR (in this case the 5-HT_1A/2A_ receptors), it undergoes a conformational change by shedding the α-subunit, resulting in the activation of the associated G-protein (Fig. 2A). Continuous receptor stimulation leads to desensitization through phosphorylation of the C-terminus of the receptor-mediated by G-protein-coupled kinases (GRKs), increasing the receptor’s affinity of the receptor for β-arrestins, which translocate to the membrane. Latter upon coupling to the GPCR complex, β-arrestins, block G-protein activation (Fig. 2B). Finally, the binding of β-arrestin, clathrin, and AP-2 facilitates endocytosis of the receptor, which can either be degraded or recycled to the plasma membrane (Fig. 2C and D).^[59,72]^

Due to their involvement in the internalization and signaling of GPRCs, several studies have been conducted to gain a better understanding of the interplay between these proteins. For instance, in vitro assays on HEK293 cell lines have demonstrated that both β-arrestin isoforms and serotonergic receptors, such as 5-HT_2A_, are co-localized within intracellular vesicles of neuronal populations, and that receptor recruitment by β-arrestin2 could occurs in response to 5-HT stimulation.^[63]^

In patients with mayor depression with and without treatment the regulation of β-arrestin-2 by GRK2 and 6 was studied. While patients having depression naïve to treatment there had an augmentation of GRK2 which is associated to the membrane in comparison with their respective healthy control individuals without any significant change in GRK6 expression.^[73]^ This augmentation was absent in patients treated with any conventional pharmacological strategy.^[73]^

This could be explained at least in part due GRK6 is almost not influenced by G serotoninergic protein coupled receptors, since internalization mediated by this enzyme is directly related to the agonism by serotonin in a homologous desensibilization process, rather than heterologous desensibilization, which for its part, could be mediated by activation of other receptors within the same cell being mediated by phosphorylation of receptors by proteins like PKC, that being not so selective could affect serotoninergic ones as well.

It is worth noting that the subtype of GRK is related and responsible for endocytosis mediated by clathrins as abovementioned^[60]^ particularly GRK2 have been proved the most efficient in clathrin recruitment, although GRK5 and GRK6 have been of relevance in this process.^[60,70]^

### 5.1. β-arrestins functional selectivity canonical and non-canonical pathways from serotoninergic receptors on depressive disorders

Activation of 5-HT G protein-coupled receptors (GPCRs) by different agonist could triggers different types of cellular signaling. Whereas serotonin activation usually leads to G protein signaling that could be influenced by β-arrestin dependent or independent internalization, diverse agonist/antagonists could have different impact and responses in different pathways. On the one hand G protein-dependent response is generally fast and transient, while the G protein-independent response, is characterized by slower onset but longer duration.^[74]^

Studies have reported the involvement of the 5-HT_1_ subfamily in depressive processes; however, the signaling mechanisms have not been fully elucidated. In this regard, various research groups such as, Liu and his working group (2019) report involvement in different signaling pathways downstream of the 5-HT_1B_ receptor. This subfamily, by coupling to Gαi/o proteins, activate MAP kinases and inhibits AC. Administration of the agonist CP-94253 in the 5-HT_1B_ cell line Neuro2A demonstrated that ERK1/2 is activated downstream and phosphorylated by the action of β-arrestin2. Notably, β-arrestin2 knockout mice do not exhibit ERK1/2 activation in response to 5-HT_1B_ receptor activation.^[75]^ There has been described different pathways to trigger β-arrestin signaling, independently from G protein activation. In particular regarding the subtype 5-HT_1C_ by calmodulin binding to the C-terminus of the receptor, preventing G-protein-mediated signaling has been demonstrated on human embryonic kidney cells (HEK 293).^[74]^

Activation of signaling cascades downstream of different signaling pathways, also modulated by β-arrestins, has been demonstrated. Several studies report that acute administration of 5-HT_1A_ receptor agonists, both administered orally (30 minutes and 60 minutes prior to assessment, respectively), increases 5-HT availability, decreases cAMP and intracellular Ca^2+^ release, as well as influences β-arrestin functionality at the cell membrane.^[61,76,77]^ Sałaciak et al^[77]^ identified that acute administration of Xanthone, a 5-HT_1A_ and 5-HT_2A_ receptor agonist, activates G-protein coupled 5-HT_1A_ and 5-HT_2A_ receptors, leading to the translocation of β-arrestin2 to the membrane. This action blocks receptor signaling to the G-protein, resulting in desensitization and subsequent internalization. These findings strongly support the involvement of β-arrestin2 in the 5-HT signaling pathway on 5-HT_2A_ receptors.

In neuronal cell lines lacking β-arrestin2, it has been observed that the 5-HT_2A_ receptor is localized solely at the soma membrane.^[78]^ Whereas, in 5-HT-treated animals, the 5-HT_2A_ receptors of cortical neurons are internalized. Moreover, mice lacking the gene for β-arrestin2 display a reduction in the number of internalized 5-HT_2A_ receptors after 5-HT administration compared to wild-type mice (with increased receptor internalization), indicating an association between β-arrestin2 and internalization of 5-HT_2A_ receptors.^[79]^ It would be very interesting to asses in which extent the β-arrestin2 is capable of deterring the signaling for this receptor alone, given a basal serotoninergic tone or if it sensitivity could be modulated by this proteins in order to eliminate excessive reactivity to the endogenous 5-HT. This would greatly reduce the undesirable effects of some antidepressants and would be an excellent complementary pharmacological strategy whereas as aforementioned biased agonism could be a promising strategy as it seems to be with another subtypes.^[61]^

On this very subtype of receptor, it is interesting to notice that is the main responsible of response generated by hallucinogenic drugs.^[80]^ In a behavioral level (at least speaking of head twitches measured) their activation remained independent from β-arrestin2 mediated pathways, this was proved by having the arrestin-3 gene knocked, and even though the head jerks in mice that were induced by DOI administration were present for wild type animals and knock outs.^[81]^ Nevertheless, it has also been demonstrated that β-arrestins are unnecessary for DOI-induced receptor internalization in mouse embryonic fibroblast, neither β-arrestin2 are required for DOI-induced ERK1/2 activation in the frontal cortex.^[82]^ In the same context of 5-HT_2A_ receptors the antagonist clozapine administration has induced internalization of them without activating ERK1/2 in the same model, as well as the prefrontal cortex of wild type mice.^[79]^ All together this evidence summarizes the complexity of determining the properties of antidepressant drugs, since the involvement of serotoninergic pathways and β-arrestins does not necessarily find the same outputs. Even when works on the same very subtype receptors differ in diverse cellular lines and highly depend on them and the zone where they are expressed on in vivo studies with will type or knock-out rodents.^[64,81]^

Regarding this receptor subfamily_,_ a very recent work by Gupta and colleges have identified a potential pocket site of union in the Ser281 and ser 295 of the intracellular loop region of the 5-HT_2B_ receptor for β-arrestin1. Although using only an *in silico* approach this could help to explain the modulation of the internalization of this receptor by said molecule and gives insights on biased agonism explanations for this receptor.^[83]^ For its part the group of Sniecikowska, have incorporated interesting evidence for the biased agonism in another receptor of this subfamily the 5-HT_1A_, by studying how the biased agonism on said serotoninergic is preferably generating an ERK1/2 signaling or a β-arrestin one. Surprisingly their results point out that β-arrestin mediated signaling was correlated with side effects of some antidepressant drugs, and for its part, when the ERK1/2 signaling was favored by the functional selectivity of the receptor, the desirable antidepressant behavior was achieved.^[61]^

According to previous studies conducted by Béchade, high levels of expression of the 5-HT_2B_ receptor were found in the hippocampus of mice, revealing the involvement of this receptor in the development and treatment of depressive-type mental disorders.^[84]^ A study using a murine chronic stress model of hopelessness (with a chronic administration of FLX 10 mg/kg) indicate that, downstream, β-arrestin2 is the signaling pathway through which the 5-HT_2B_ receptor inhibits A1 astrocyte activation in the hippocampus.^[85]^ These reports demonstrate the interaction between β-arrestin2 expression and the response to antidepressant drug, suggesting that β-arrestin2 not only regulates the desensitization-internalization of GPCRs, but also plays a role in the regulation of downstream signaling pathways in the hippocampus. Results indicate that the inhibition of FLX on the activation of astrocyte A1 is independent of the Gq protein or β-arrestin1 in vitro, and that the activation of β-arrestin2 is responsible for carrying out the signaling in chronic stress models.^[85]^

In fact, there are very interesting works regarding 5-HT_2c_ receptors as well, published by Labasque et al.^[86]^ In 2010 this group demonstrated on a very elegantly manner, employing HEK293 cells, that these receptors had a constitutive activity, which instead of being conducted by a G protein canonical pathway, was dependent on ERK1/2 signaling, given the fact that this activity was abolished with a siRNA capable of deterring the activity of β-arrestin, thus emphasizing the relevance of the functional selectivity of the receptors. The effects of deterring the activity of ERK1/2 were blocked by administration of tricyclic antidepressant but not by FLX thus suggesting biased agonism by these different types of drugs.

Another group have very recently found that 5HT2c receptor in a heterodimeric structure formed along with oxytocin receptor, found probable that their specific behavioral differential response could be mediated by this construct, and that β-arrestin could play a role in its formation and further signaling in a in vivo approach (Chruscicka, Cowan et al 2021).

The 5-HT_7_ receptor has recently been described as a target, and its inverse agonism demonstrates different effects mediated by a β-arrestin2-dependent mechanism of ERK1/2 activation. Although its effects have only been associated with pain reduction, it remains to be explored whether these pharmacological manipulations could potentially lead to antidepressant effects in the future.^[56]^ The signaling pathways linked to stress response, anxiety, and depressive disorders, cell differentiation and migration, activation of proinflammatory processes, and neuronal plasticity are crucial in responding to drugs such as antidepressants, the action of various neurotransmitters, and the brain homeostasis.^[87]^

**Figure 3.**
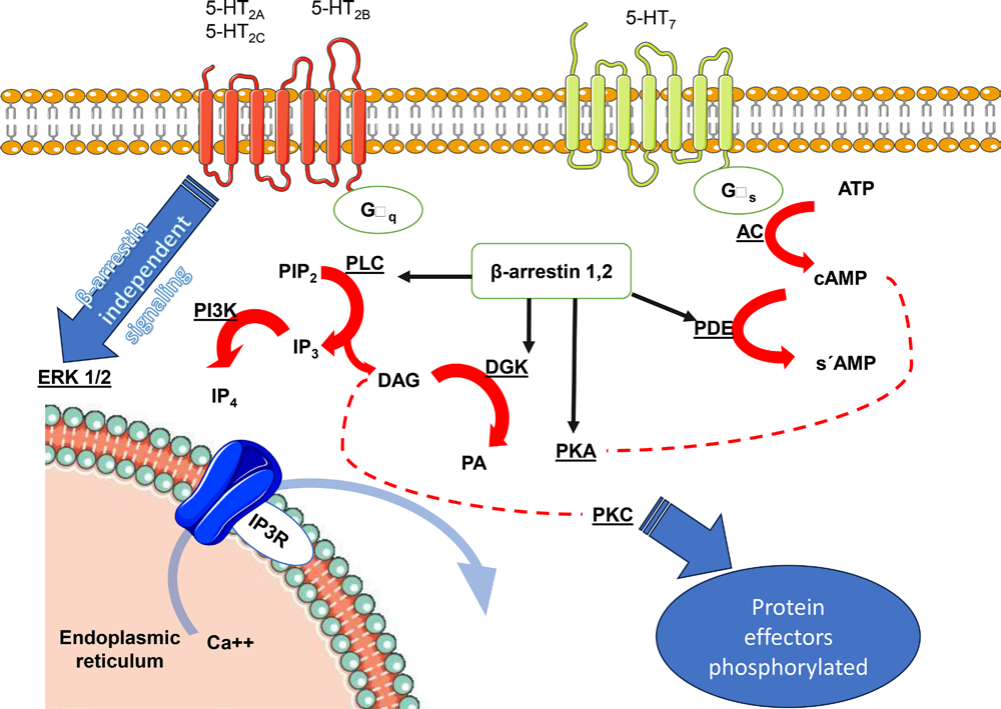
Comparative signaling of 5-HT Gq and Gs receptors. For each type of G protein coupled receptor, the canonical signaling and some interactions involving β-arrestins with diverse second messengers are shown. On the one hand β-arrestin could promote independent signaling regardless of G protein signaling, this has been observed more frequently with extracellular regulated kinases ERK1/2 pathway as well as protein kinase B (Akt). On the other hand, these proteins could be modifying (reducing or amplifying) the signaling that has already been initiated by G protein canonical signaling. Non continuous lines represent indirect influence. Protein kinase A, C, (PKA, PKC), Phospholipase C (PLC), (Phosphatidyl) Inositol biphosphate, triphosphate, tetraphosphate, receptor (P) IP 2,3,4, R, AC = adenyl cyclase, ATP = adenosine triphosphate, cAMP = cyclic adenosine monophosphate, DAG = diacyl glycerol, DGK = diacyl glycerol kinase, PDE = phosphodiesterase.

**Figure 4.**
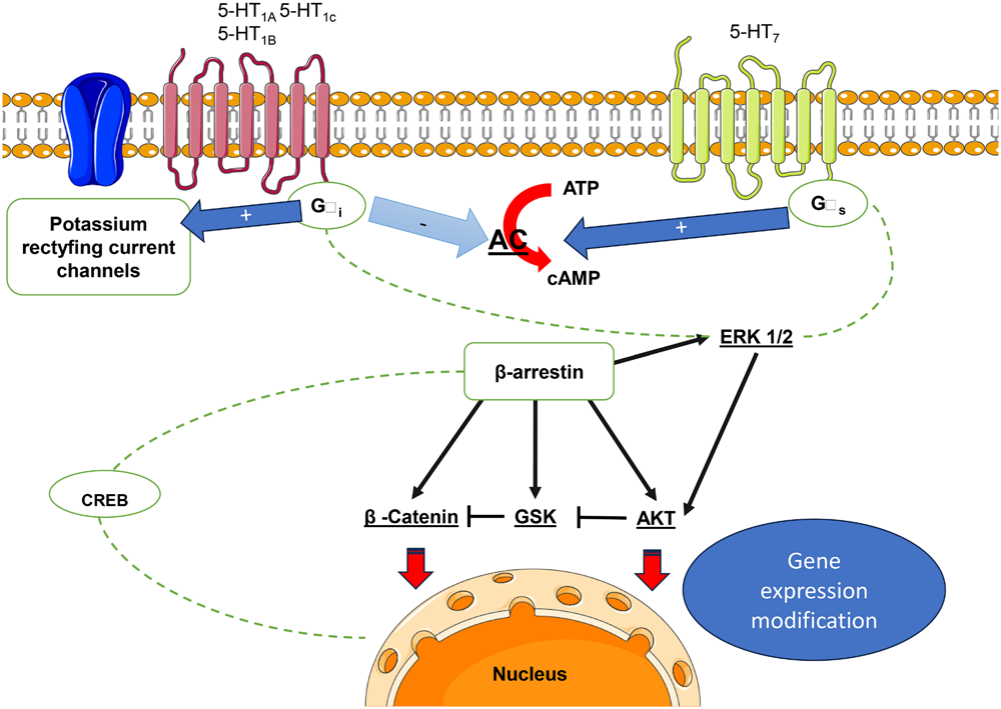
Comparative signaling of 5-HT Gi and Gs receptors. For each type of G protein coupled receptor, the canonical signaling and some interactions involving β-arrestins with diverse second messengers are shown. On the one hand β-arrestin could promote independent signaling regardless of G protein signaling, this has been observed more frequently with extracellular regulated kinases ERK1/2 pathway as well as protein kinase B (Akt). On the other hand, these proteins could be modifying (reducing or amplifying) the signaling that has already been initiated by G protein canonical signaling. Non continuous lines represent indirect influence. Protein kinase A, C, (PKA, PKC), Phospholipase C (PLC), (Phosphatidyl) Inositol biphosphate, triphosphate, tetraphosphate, receptor (P) IP 2,3,4, R. AC = adenyl cyclase, ATP = adenosine triphosphate, cAMP = cyclic adenosine monophosphate, DAG = diacyl glycerol, DGK = diacyl glycerol kinase, PDE = phosphodiesterase.

Depending on the conformational change of the receptor, β-arrestins has been shown to play an important role in the regulation and activation of downstream signaling pathways such as NF-κB, ERK1/2, MAPK, and AMPK/mTOR.^[7,88,89]^ These and other mechanisms previously discussed are presented in (Figs. 3 and 4), illustrating the relationship between β-arrestin and the serotoninergic system mainly in depressive disorders. Meanwhile there is not a vast list of recent papers regarding this, some new insights could be found in literature.^[2,56,58,61,83,85]^

Regarding β-arrestins 1 and 2 on their own, they have been measured in patients with and without depression, before and after the treatments as well as their level of ubiquination. The first thing to notice is that β-arrestins were lower in the mononuclear limphocytes of patients versus their healthy controls. And these changes were reversed to normality when patients were treated. Whilst untreated patients presented less ubiquitination of both β-arrestins when compared to healthy people treatment was also capable of raise β-arrestin2 ubiquitination without modifying this process in the other protein.^[67,90]^ This was also observed in the same group of researchers in another cohort of patients and an animal model where β-arrestin2 was diminished along with G proteins in the periphery pointing out the importance of the interplay between these proteins with the depressive disorders.^[67]^

## 6. Conclusion

These findings highlight the significant role of serotonergic receptors in depressive disorders, emphasizing the importance of their modulation in treating depression. Furthermore, investigations into response mechanisms have revealed β-arrestins as an important modulator of diverse signaling pathways, ranging from a regulator in the endocytosis process, to an activator of cellular cascades independent to the canonical G protein signaling. The variations on the responses could not be generalized, due to significant differences between diverse receptors subtypes, as well as contrasting results seen under in vitro versus in vivo approaches. The biased agonism, favoring one signaling pathway or another as we have seen, could be bringing potentially beneficial effects against depressive symptoms. Although the evidence in the literature remains inconclusive regarding whether is β-arrestin signaling better or worse against depressive disorders. More than a dichotomic answer, the most adequate point of view, will be based on the circumstances revolving one particular experiment or set of experiments. Given a plethora of factors that could change the response substantially, such as the model used to study, the specific drugs employed and even the methodologies for measuring results, there is not a simple answer. A similar situation is seen with the endocytosis of serotoninergic receptors, it is not possible to simply say that is beneficial or detrimental for depressive conditions. This depends on which specific subtypes are being endocyted, at what extent, and in which cellular stirpes and sub-regions this process is occurring. There is still a lot to unveil regarding the mechanisms that pharmacology could take advantage of in the functional selectivity field involving β-arrestins and serotoninergic receptors. This biased agonism strategy although could be capable of alleviating mood disorders and symptoms in certain scenarios, is still in great debate because there are limitations on the understanding of it as a general process. Although these features, difficult the possibility to claim the targeting of β-arresitins as a universally effective pharmacological approach, it could be promising to selectively treat or improve certain pathologic conditions such as depressive disorders given the close relationship between the serotoninergic system with these mediators.

## Author contributions

**Conceptualization:** Mario E. Flores-Soto.

**Writing – original draft:** Aldo R. Tejeda-Martínez, Ana R. Ramos-Molina.

**Writing – review & editing:** Aldo R. Tejeda-Martínez, Patricia A. Brand-Rubalcava, Mario E. Flores-Soto.
